# Maximising and Maintaining Fertility in Suspected or Confirmed Endometriosis: A Structured Narrative Review for Clinical Practice

**DOI:** 10.7759/cureus.112350

**Published:** 2026-07-09

**Authors:** Mouli Nandi, Rana Mondal

**Affiliations:** 1 Department of Obstetrics and Gynaecology, Luton and Dunstable University Hospital, Luton, GBR

**Keywords:** assisted reproduction, endometrioma, endometriosis, fertility preservation, infertility, oocyte cryopreservation, ovarian reserve, reproductive surgery

## Abstract

Endometriosis affects a substantial proportion of women of reproductive age and is a common, often under-recognised contributor to subfertility. Its effects on fertility are multifactorial, and both the disease and its treatment, particularly ovarian surgery, can reduce ovarian reserve. Clinicians are frequently required to balance pain control, restoration of anatomy, assisted reproduction, and the protection of future fertility, often with imperfect evidence. The objective of this review was to synthesise current evidence and guideline recommendations on the interventions and care pathways that most effectively maximise the chance of pregnancy while minimising avoidable loss of ovarian reserve for women who are trying to conceive and those wishing to preserve future fertility. We searched PubMed/MEDLINE (Medical Literature Analysis and Retrieval System Online), the Cochrane Library and, where accessible, Embase and Scopus, from January 2010 to June 2026, supplemented by landmark earlier trials and by guidelines from the European Society of Human Reproduction and Embryology (ESHRE), National Institute for Health and Care Excellence (NICE), and American Society for Reproductive Medicine (ASRM). Clinical guidelines, systematic reviews, meta-analyses, randomised controlled trials and large prospective cohort studies addressing endometriosis, ovarian reserve, endometrioma surgery, assisted reproduction and fertility preservation were prioritised. This was a structured narrative review and not a systematic review; no formal risk-of-bias scoring or quantitative meta-analysis was undertaken.

Age, ovarian reserve, and duration of infertility are the dominant prognostic factors and should drive timing. Excisional surgery for minimal-to-mild disease produces a modest absolute increase in spontaneous conception, whereas ovarian endometrioma cystectomy consistently lowers anti-Müllerian hormone and is the principal iatrogenic threat to ovarian reserve. Assisted reproduction outcomes in women with endometrioma are broadly comparable to those without, and surgery before in vitro fertilisation does not reliably improve live birth; routine cystectomy before treatment is therefore not supported. Medical hormonal therapy controls pain and reduces recurrence, but suppresses ovulation and is not a fertility treatment during attempts to conceive. Oocyte and embryo cryopreservation are reasonable options before bilateral or repeat ovarian surgery and for women delaying conception, but success is age-dependent and not guaranteed. Emerging or investigational approaches-oral gonadotrophin-releasing hormone antagonists, reserve-sparing ethanol sclerotherapy of endometrioma, the Endometriosis Fertility Index as a counselling tool, intraovarian platelet-rich plasma and artificial-intelligence-assisted imaging-are reviewed with appropriate caution. Fertility should be discussed early, at suspicion, not only at confirmation. An individualised pathway that protects ovarian reserve, avoids unnecessary or repeated ovarian surgery, integrates assisted reproduction without delay in higher-risk women, and offers timely fertility preservation is most likely to maximise reproductive outcomes. Access inequities make pragmatic, resource-sensitive pathways a global priority.

## Introduction and background

Endometriosis, the presence of endometrial-like tissue outside the uterus, affects an estimated one in 10 women of reproductive age and is identified in a substantially higher proportion of women investigated for subfertility [1]. It is a chronic, oestrogen-dependent inflammatory condition, the relation of which to fertility is complex and incompletely understood. Many affected women conceive without difficulty; others experience profound subfertility. Because the condition is common, frequently diagnosed late, and frequently coexists with the very interventions that can themselves reduce fertility, it occupies a central and sometimes uncomfortable position in reproductive medicine [1-4].

The mechanisms by which endometriosis impairs fertility are multiple and often coexist. Advanced disease distorts pelvic anatomy through adhesions and fibrosis, impairing ovum pick-up and tubal transport. Ovarian endometriomas may reduce the volume of healthy cortex and the density of follicles, and are associated with lower ovarian reserve even before any surgical intervention. The peritoneal environment in endometriosis is characterised by chronic inflammation, with altered cytokine and macrophage activity that may impair oocyte quality, fertilisation, sperm function and early embryo development. Folliculogenesis and oocyte competence may be compromised, endometrial receptivity may be altered through progesterone resistance and aberrant gene expression, and tubal function may be affected even when the tubes appear patent. Coexisting adenomyosis can further reduce implantation and increase early pregnancy loss. Finally, dyspareunia and chronic pelvic pain may reduce the frequency of intercourse, and ovarian surgery, however carefully performed, can remove or damage functional ovarian tissue [1-4].

The principal mechanisms by which endometriosis impairs fertility, their clinical implications, and the interventions that may modify them are summarised in Table 1.

**Table 1:** Fertility-impact mechanisms in endometriosis AMH, anti-Müllerian hormone; AFC, antral follicle count; GnRH, gonadotrophin-releasing hormone; ICSI, intracytoplasmic sperm injection; IUI, intrauterine insemination; IVF, in vitro fertilisation References: [1,4]

Mechanism	Effect on fertility	Clinical implication	Modifiable intervention
Chronic peritoneal inflammation	Altered cytokine/macrophage activity may impair oocyte quality, fertilisation, and early embryo development	Subfertility may persist despite normal anatomy	Assisted reproduction (IVF/ICSI) bypasses much of the hostile environment
Adhesions / anatomical distortion	Impaired ovum pick-up and tubal transport	Reduced spontaneous conception	Adhesiolysis and restoration of anatomy; IVF if access remains compromised
Ovarian endometrioma	Reduced healthy cortex and follicle density; lower reserve even before surgery	Lower oocyte yield; surgery may worsen reserve	Conservative management; fertility preservation; selective surgery
Surgical ovarian injury	Loss of cortex and thermal damage lower AMH/AFC, especially after bilateral or repeat surgery	Iatrogenic reduction in reserve	Fertility-sparing technique; minimise diathermy; avoid unnecessary/repeat surgery; preserve before surgery
Tubal dysfunction	Impaired transport even when tubes appear patent	Reduced natural and IUI success	IVF where tubal function is in doubt
Impaired folliculogenesis / oocyte competence	Possible reduction in oocyte and embryo quality	Lower pregnancy rate per cycle	No reliable direct modifier; optimise timing and reserve
Altered endometrial receptivity	Progesterone resistance and aberrant gene expression may impair implantation	Implantation failure	Limited; individualised assisted-reproduction strategies
Coexisting adenomyosis	Reduced implantation and increased early pregnancy loss	Lower IVF success; higher miscarriage	Consider GnRH-agonist down-regulation or freeze-all (limited evidence)
Dyspareunia / chronic pain	Reduced frequency of intercourse	Reduced natural conception	Effective pain control; timed intercourse; assisted reproduction
Delayed diagnosis	Disease progression and ageing before treatment	Narrowed reproductive options	Earlier recognition, referral and counselling

These mechanisms have an important clinical corollary: opportunities to protect fertility arise long before a woman presents with established infertility. A young woman with cyclical pain and an ovarian endometrioma identified on ultrasound, who is not yet trying to conceive, faces decisions about surveillance, surgery, and fertility preservation that may determine her future reproductive options. For this reason, fertility counselling should begin at the point of suspicion or diagnosis, not be deferred until repeated surgery or advancing age has narrowed the available choices [5].

The topic is of global importance. Diagnostic delay, frequently several years from symptom onset, is a near-universal problem and is compounded in many settings by limited access to specialist imaging, high-quality endometriosis surgery, assisted reproduction, and fertility preservation. A review aimed at an international readership must therefore offer guidance that is clinically sound where resources are abundant, and pragmatic where they are constrained. This structured narrative review summarises current evidence and guideline recommendations on how best to maximise the chance of pregnancy for women trying to conceive at present and for those wishing to preserve future fertility, while minimising avoidable loss of ovarian reserve. It also surveys newer and investigational management options, distinguishing carefully between established practice and approaches that remain experimental.

## Review

Methods: search strategy and evidence synthesis

We conducted a structured literature search to inform a narrative synthesis. PubMed/MEDLINE (Medical Literature Analysis and Retrieval System Online) and the Cochrane Library were searched systematically, and Embase, Scopus, and Web of Science were consulted where institutional access allowed. The principal search period was January 2010 to June 2026, with landmark earlier trials retained where they remain clinically influential, such as the randomised trial of laparoscopic surgery for minimal-to-mild endometriosis [5]. Guidelines from the European Society of Human Reproduction and Embryology (ESHRE), the National Institute for Health and Care Excellence (NICE), and the American Society for Reproductive Medicine (ASRM) were reviewed directly from the issuing bodies [1-4].

Search terms combined the core concepts of endometriosis (including endometrioma and deep endometriosis) and adenomyosis with terms for infertility, fertility preservation and ovarian reserve (anti-Mullerian hormone (AMH) and antral follicle count (AFC)), surgical intervention (cystectomy, ablation and ethanol sclerotherapy), assisted reproduction (in vitro fertilisation (IVF), intracytoplasmic sperm injection (ICSI), and intrauterine insemination (IUI)), oocyte and embryo cryopreservation, gonadotrophin-releasing hormone (GnRH) agonists and antagonists, dienogest, disease recurrence and postoperative suppression, combined using the Boolean operators AND and OR. We prioritised clinical guidelines, systematic reviews and meta-analyses, randomised controlled trials, and large or high-quality prospective cohort studies, and screened the reference lists of key articles for additional sources.

This was a structured narrative review and not a systematic review: no protocol was registered, no duplicate screening or formal risk-of-bias scoring was undertaken, and no Preferred Reporting Items for Systematic reviews and Meta-Analyses (PRISMA) flow diagram was generated. Evidence was synthesised narratively rather than pooled quantitatively, reflecting the substantial clinical and methodological heterogeneity of the field: studies differed in disease phenotype and stage, surgical technique and surgeon experience, ovarian stimulation protocols, and outcomes reported, which ranged from biochemical markers of reserve through clinical pregnancy to live birth. The strengths and limitations of the underlying evidence are stated explicitly throughout.

The recommendations in this review are organised around a stepwise clinical sequence: first, age and fertility intention were assessed; second, ovarian reserve were assessed using AMH and AFC; third, the disease phenotype was defined, including endometrioma laterality, deep endometriosis, adenomyosis, symptoms and previous surgery; fourth, it was determined whether there was a clear surgical indication; and finally, the least harmful fertility-directed pathway was selected: conservative management, surgery, assisted reproduction first, or fertility preservation first. This sequence was intended to make the review clinically usable at the point of counselling and to avoid reflexive or repeated ovarian surgery where it is unlikely to improve fertility.

Initial assessment, fertility intention, and baseline prognosis

The single most important determinant of reproductive prognosis is female age, and no intervention discussed in this review reverses age-related decline in oocyte quality and quantity. Counselling and decision-making should therefore be anchored in age, alongside duration of infertility, symptom severity and disease extent [3,4]. A woman aged 31 years with a small, asymptomatic endometrioma and a short history occupies a very different prognostic position from a woman aged 39 with bilateral endometriomas and three years of infertility, and the pathways that serve them best diverge accordingly.

Assessment of ovarian reserve before choosing treatment

Ovarian reserve should be assessed with serum AMH and AFC, with an understanding of their limitations. Both markers predict the quantity of the ovarian response to stimulation better than they predict the chance of natural conception or the quality of individual oocytes; neither should be used in isolation to counsel a woman that pregnancy is impossible, nor to justify aggressive intervention. AMH assays vary between laboratories, and values can fluctuate; a single low result warrants confirmation and cautious interpretation, particularly in younger women. Nonetheless, a low or falling AMH, especially before planned ovarian surgery, is a powerful argument for early specialist referral and for considering fertility preservation [6,7].

A complete baseline assessment also considers tubal status where clinically relevant and, crucially, the male partner. Semen analysis is inexpensive, modifiable in its implications, and too often deferred; a significant male factor changes the entire pathway and may move a couple directly towards IVF with ICSI. Coexisting conditions should be sought and documented: adenomyosis, fibroids, polycystic ovary syndrome, tubal disease, and the legacy of previous pelvic or ovarian surgery each modify prognosis and management [3,4]. In surgically staged disease, the validated Endometriosis Fertility Index (EFI) can help predict the likelihood of non-IVF conception and guide whether to attempt natural conception or move to assisted reproduction; it can now be estimated reasonably well from clinical and ultrasound information before any operation [8-14].

These elements should feed into genuine shared decision-making, in which the woman's reproductive intentions, values, and circumstances are explicit and documented. The central message is preventive: fertility planning should not wait until severe disease, diminished reserve, or repeated surgery has occurred. Early, honest counseling, even when conception is not yet being attempted, is itself one of the most effective fertility-protecting interventions available. Table 2 is intended as a clinical decision support tool rather than a rigid algorithm. It should be used sequentially: first to identify the woman’s reproductive goal, then to assess ovarian reserve and disease-related risk, and finally to select the least harmful pathway that preserves fertility while addressing symptoms and safety concerns (Figure 2). Table 3 summarizes the individual strategies to maximize or maintain fertility, with their best candidates and the strength of the supporting evidence.

**Table 2:** Clinical pathway according to reproductive goal. ART, assisted reproductive technology; MDT, multidisciplinary team; MRI, magnetic resonance imaging; US, ultrasound References: [1,3,4,9,10]

Clinical Scenario	First Assessment	Preferred Approach	When to Refer to a Fertility Specialist	Key Caution
Not currently trying to conceive	Age, symptoms, imaging, baseline AMH/AFC	Counselling; surveillance; pain control; consider preservation if reserve is at risk	Before any planned ovarian surgery, if reserve low or disease severe	Do not let the opportunity to preserve reserve pass unaddressed
Trying to conceive <12 months	Age, duration, semen analysis, reserve	Expectant management with timed intercourse if favourable features	If age ≥35, low reserve, or adverse features	Avoid premature surgery; set review point
Infertility ≥12 months (or ≥6 months if age ≥35)	Full couple assessment incl. tubal and male factor	Assisted reproduction (IUI for minimal/mild + patent tubes; otherwise IVF/ICSI)	Refer now	Do not delay; each cycle of waiting has a cost
Endometrioma before planned surgery	Reserve (AMH/AFC), laterality, prior surgery	Discuss fertility preservation before operating; confirm indication	Before surgery	Bilateral/repeat surgery threatens reserve—preserve first
Endometrioma before IVF	Reserve, cyst size, access for retrieval, symptoms	Proceed to IVF without routine cystectomy	Refer for IVF	Surgery does not improve IVF live birth and may reduce yield
Deep endometriosis	Symptoms, anatomy (US/MRI), age, reserve, access to ART	Individualised: ART-first or specialist surgery; MDT	Early, alongside specialist centre	Surgery not proven to improve live birth in all cases
Recurrent endometrioma after previous surgery	Reserve, prior operative detail, symptoms	Favour conservative/ART; avoid repeat surgery unless clearly indicated	Refer before any re-operation	Repeat surgery risks premature ovarian insufficiency
Diminished ovarian reserve	Confirm AMH/AFC; age; partner	Expedited assisted reproduction ± preservation	Refer urgently	Avoid ovarian surgery; time is critical

**Figure 1:**
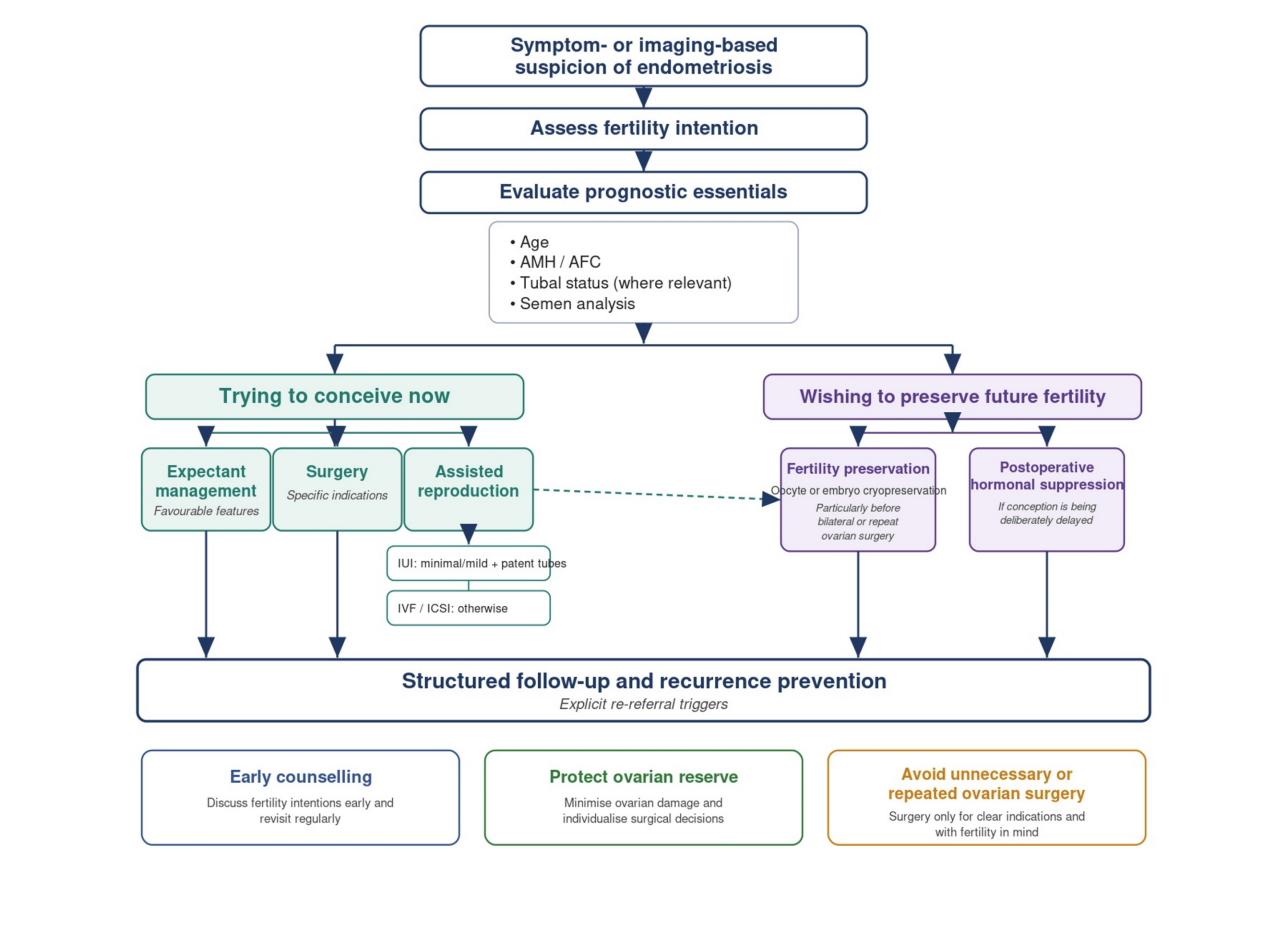
Flowchart showing fertility-preserving care pathway for suspected or confirmed endometriosis. The figure emphasises early counselling, protection of ovarian reserve, and avoidance of unnecessary or repeated ovarian surgery. IVF, in vitro fertilisation; ICSI, intracytoplasmic sperm injection; IUI, intrauterine insemination; AMH: anti-Mullerian hormone; AFC: antral follicle count Image Credit: Authors; created using Adobe Illustrator (Adobe Inc., San Jose, California, United States)

**Table 3:** Strategies to maximise or maintain fertility. ‘Emerging/investigational’ strategies are not yet recommended for routine use. RCT, randomised controlled trial; IVF, in vitro fertilisation; ICSI, intracytoplasmic sperm injection; IUI, intrauterine insemination; GnRH, gonadotrophin-releasing hormone; AMH: anti-Mullerian hormone; AFC: antral follicle count Compiled by the authors from references [8-14].

Strategy	Best Candidates	Main Benefit	Main Limitation/Risk	Strength of Evidence
Early fertility counselling	All women with suspected/confirmed disease	Preserves options; informs timing	Requires awareness and access	Expert consensus / guideline-supported
Ovarian reserve testing (AMH/AFC)	Those facing surgery or delay	Triage and timing inform preservation	Predicts quantity, not quality; assay variability	Moderate
Expectant management	Young, short history, mild disease, good reserve	Avoids surgery and ART cost/risk	Wasted time if prognosis poor	Moderate
Laparoscopic treatment of minimal/mild disease	Stage I–II, trying to conceive	Modest increase in spontaneous conception	Operative risk; modest absolute benefit	Moderate (RCT + meta-analysis)
Endometrioma cystectomy	Pain, suspicious features, access problems	Lower recurrence; symptom relief; spontaneous pregnancy in subfertile	Significant fall in AMH; worse if bilateral/repeat	Strong (reserve harm); moderate (fertility)
Endometrioma ablation/laser/plasma	Reserve protection a priority	Better reserve preservation than excision	Higher recurrence than excision	Low–moderate/conflicting
IVF/ICSI	Moderate–severe disease, tubal/male factor, age, low reserve, failed prior steps	Effective; outcomes near those without disease	Cost; access; cycle cancellation higher with endometrioma	Strong
IUI with ovarian stimulation	Minimal/mild disease, patent tubes, no male factor	Less invasive/costly first step	Lower per-cycle success than IVF	Moderate
Oocyte cryopreservation	Before ovarian surgery; delaying conception; severe disease	Banks gametes while reserve adequate	Age-dependent; fewer oocytes with endometrioma; no guarantee	Moderate
Embryo cryopreservation	Women with a partner/donor sperm	Established efficacy	Requires sperm; legal/relationship considerations	Moderate–strong
Postoperative hormonal suppression (if delaying conception)	Not conceiving immediately after surgery	Reduces recurrence; protects interval	Suppresses ovulation—must not delay conception when ready	Moderate
Avoidance of repeat ovarian surgery	Recurrent endometrioma, prior cystectomy	Protects remaining reserve	Requires symptom control by other means	Moderate / consensus
Oral GnRH antagonist (relugolix/elagolix): emerging	Pain; wish to defer reserve-damaging surgery	Effective pain control; may reduce need for surgery	Ovulation-suppressing (not a fertility treatment); bone density	Strong for pain; indirect for fertility
Ethanol sclerotherapy of endometrioma: emerging	Endometrioma before IVF; reserve protection	Minimally invasive; AMH-sparing; possibly more oocytes	Not standardised; recurrence; investigational	Low–moderate / emerging
Intraovarian platelet-rich plasma: investigational	Diminished reserve (research settings only)	Possible modest improvement in reserve markers	Preliminary, largely uncontrolled data	Experimental

Define the disease phenotype and diagnostic certainty

Management should be calibrated to diagnostic certainty as well as to fertility goals. It is useful to distinguish four situations. The first is suspected endometriosis based on symptoms (cyclical pelvic pain, dysmenorrhoea, dyschezia, dyspareunia) with or without suggestive but non-diagnostic imaging. Here, fertility counselling and reserve assessment can and should proceed without waiting for surgical confirmation; laparoscopy purely to confirm a diagnosis is no longer required before managing subfertility [1,2].

The second is imaging-confirmed disease, an endometrioma with characteristic ground-glass echogenicity on transvaginal ultrasound, or features of deep endometriosis on ultrasound or MRI. Imaging confirmation allows planning but does not in itself mandate surgery. The third is surgically and histologically confirmed disease, which provides the most definitive diagnosis and staging, but at the cost of an operation that may have already affected ovarian reserve. The fourth is incidental or asymptomatic endometriosis, discovered during imaging or surgery for another indication; in the absence of symptoms or a fertility problem, intervention is rarely warranted, and the priority is counselling and surveillance.

Across these categories, the practical point is that a confident diagnosis is not a prerequisite for protecting fertility. Women with suspected disease deserve the same early conversation about age, reserve, and reproductive intentions as women with histological confirmation and may stand to benefit most, because their options are still open. Advances in high-resolution ultrasound, structured imaging classifications and, more recently, artificial intelligence (AI)-assisted image interpretation may in time improve non-invasive diagnosis, although current automated tools remain investigational and have not yet matched expert sonography [15].

Expectant management

For appropriately selected couples, a period of expectant management with timed regular intercourse is reasonable and avoids both the risks of surgery and the costs of assisted reproduction. Favourable features include younger age, short duration of infertility, mild symptoms, no major anatomical distortion, good ovarian reserve, the absence of a significant male factor, and either no endometrioma or a small, stable, asymptomatic one. A favourable EFI score supports this approach, and patient preference, after balanced counselling, is a legitimate and important part of the decision [16,17].

Expectant management is inappropriate, or should be firmly time-limited, when prognosis is poorer or when delay carries a measurable cost to future fertility. Advanced reproductive age, diminished ovarian reserve, bilateral endometriomas, severe pain, deep disease affecting bowel or ureter, hydrosalpinx or other significant tubal disease, previous ovarian surgery, and prolonged infertility all argue against simply waiting. In such situations, the opportunity cost of each month of expectant management is real, and earlier movement towards assisted reproduction-or towards fertility preservation if conception is not yet desired-is usually the safer course [1,3,4]. The clinician's role is to set explicit time limits and review points, rather than to allow expectant management to drift indefinitely.

Medical therapy and its place in fertility planning

A recurrent source of confusion, for clinicians and patients alike, is the role of hormonal therapy. Combined oral contraceptives, progestins (including dienogest), the levonorgestrel-releasing intrauterine system (LNG-IUS), gonadotrophin-releasing hormone (GnRH) agonists and antagonists, and aromatase inhibitors are effective for pain and can reduce recurrence. None of them enhances fertility while being taken: by suppressing ovulation, they prevent conception during use. There is no good evidence that a course of ovarian suppression improves spontaneous fertility once stopped, and such therapy should not be offered as a fertility treatment to women actively trying to conceive [1,4]. This distinction between treating pain and treating infertility must be made explicit because well-intentioned prescription of suppressive therapy can waste reproductive time in women whose fertility window is already narrowing.

Hormonal therapy nonetheless has a legitimate place in fertility planning. For a woman who has had surgery but is not yet ready to conceive, postoperative suppression can control symptoms and reduce the risk of disease and endometrioma recurrence during the interval before she starts trying, potentially preserving the benefit of surgery and reducing the likelihood of repeat operations [1]. Postoperative use of the LNG-IUS reduces the recurrence of painful periods, and continuous progestins such as dienogest reduce recurrence, although long-term progestin use can modestly lower bone mineral density and warrants counseling [18-21]. The evidence here concerns recurrence and symptom control rather than a direct fertility benefit, and therapy should be timed so that it does not itself delay conception in women who are ready to proceed [22,23].

The use of medical suppression, specifically before IVF, deserves a cautious note. Prolonged GnRH-agonist down-regulation before IVF has been studied and may be considered in selected circumstances, for example, where adenomyosis coexists, but the evidence base is limited and not uniformly positive, and it should not be presented as a routine fertility-enhancing step for all women with endometriosis [17,18]. Newer oral GnRH antagonists are considered in the section on emerging options below; their role to date is in pain control rather than fertility enhancement [24,25].

Surgery for fertility optimisation

Surgery in endometriosis serves several distinct purposes, and conflating them leads to poor decisions. It may be undertaken for pain relief, for restoration of distorted anatomy, to improve the chance of spontaneous conception, to improve access to or the safety of assisted reproduction, or to exclude malignancy where imaging is atypical. These indications overlap but are not identical, and the strength of evidence differs across them.

For minimal-to-mild (stage I-II) disease in women trying to conceive, laparoscopic excision or ablation of peritoneal deposits, with adhesiolysis and restoration of anatomy, produces a modest improvement in spontaneous conception. In the landmark randomised trial by Marcoux et al., operative laparoscopy increased the cumulative probability of ongoing pregnancy from approximately 18% to 31% over the months following surgery [5]; a Cochrane review that pooled this trial with a second, smaller randomised trial confirmed a significant benefit of laparoscopic surgery on pregnancy rates [26]. The effect is real but modest in absolute terms, equating to several women needing treatment for one additional pregnancy; it must be weighed against operative risk, cost and the alternative of proceeding to assisted reproduction. Adding ovulation induction after surgery increases ovulation but has not been shown to add a clear live-birth advantage over surgery alone, and where stimulation with intrauterine insemination is used, letrozole and clomiphene citrate appear comparably effective [11,12].

Surgery for deep endometriosis when fertility is the priority is more contentious. The decision to operate before natural conception rests on the likelihood that restoring anatomy will meaningfully improve the chance of pregnancy, balanced against the risk to ovarian reserve and the morbidity of complex surgery. The decision to operate before assisted reproduction is different again: because IVF bypasses much of the distorted anatomy, the case for pre-treatment surgery is correspondingly weaker, and the default should not be to operate on all disease before IVF [4,20].

Two principles apply across all surgical indications. First, complex disease, such as deep endometriosis involving bowel, bladder, or ureter, and difficult or recurrent endometriomas, should be managed in specialist centres with multidisciplinary expertise, where outcomes are better and complications fewer [27-31]. Second, where fertility is a consideration, surgical technique should be explicitly fertility-sparing, with meticulous attention to preserving ovarian tissue. Surgery undertaken primarily for pain in a woman who also wishes to conceive should still be planned with reserve protection in mind. Where imaging shows atypical, solid or rapidly enlarging features, the small but real risk of endometriosis-associated ovarian malignancy is itself a legitimate indication to operate [15,32].

Fertility-directed pathway for endometrioma

Management of the ovarian endometrioma is the area where the tension between treating disease and protecting fertility is sharpest, and it deserves detailed, nuanced consideration. Endometriomas are associated with reduced ovarian reserve even before surgery, reflecting damage to the surrounding cortex from the cyst itself and the local inflammatory milieu. Surgery then introduces a second, iatrogenic hit [1,7].

The evidence that excisional surgery reduces ovarian reserve is consistent. Meta-analyses of serum AMH before and after laparoscopic cystectomy show a significant postoperative fall, with one early synthesis reporting a weighted mean decrease of around 1.1 ng/mL and a more recent and larger meta-analysis confirming a significant decline that persists into the longer term [7,8]. The mechanism is loss of healthy cortex stripped with the cyst wall and thermal injury from haemostatic coagulation. The fall is greater after bilateral cystectomy, in which both ovaries are exposed to this damage, and repeat surgery on a recurrent endometrioma compounds the loss, occasionally to the point of precipitating premature ovarian insufficiency [8].

When excision is chosen, technique matters. Surgeons should aim to identify and develop the correct cleavage plane, strip the cyst wall with minimal removal of adjacent ovarian parenchyma, and minimise bipolar diathermy, favouring targeted haemostasis, suturing or haemostatic sealants over extensive coagulation of the ovarian bed. Operator experience is itself a determinant of reserve preservation [1,27]. Where the decision is between cystectomy and an ablative approach (CO2 laser, plasma energy, or controlled bipolar ablation of the cyst capsule after drainage), the trade-off is between recurrence and reserve. Excisional surgery is associated with lower recurrence of the endometrioma and of symptoms, and with a higher subsequent spontaneous pregnancy rate in previously subfertile women, but at greater cost to reserve; ablative and combined techniques may better preserve reserve but with a higher recurrence risk [6]. The optimal approach is therefore individualised, and ablative or combined techniques are reasonable where reserve protection is paramount. Reserve-sparing ethanol sclerotherapy, discussed under emerging options, is a further alternative under active investigation [28].

Surgery for an endometrioma may be appropriate when there is significant pain unresponsive to medical management, when imaging features are suspicious or atypical, growth is rapid (raising concern about malignancy), a large cyst impairs safe access to follicles for oocyte retrieval or causes severe anatomical distortion, or there is a meaningful risk of rupture or infection. Failure of conservative management is a further reasonable indication [1,4].

Conversely, surgery should often be avoided or deferred. An asymptomatic endometrioma in a woman about to undergo IVF should generally not be removed routinely, because cystectomy does not reliably improve IVF outcomes and may reduce the oocyte yield [9,10]. Low ovarian reserve, bilateral disease, recurrent cysts, previous ovarian surgery, and advanced age, where time cannot be spared and reserve cannot be risked, all argue against operating and in favour of proceeding directly to assisted reproduction or fertility preservation. The presence of an endometrioma is not, in itself, an indication for surgery. Table 4 summarises endometrioma decision-making before IVF or surgery across the common clinical scenarios. 

**Table 4:** Endometrioma decision-making before IVF or surgery. ART, assisted reproductive technology; POI, premature ovarian insufficiency; IVF, in vitro fertilisation Compiled by the authors from references [1,6-10,28,33,34]

Scenario	Surgery Favoured?	ART/Fertility Preservation Favoured?	Rationale	Practical Note
Asymptomatic endometrioma before IVF	No	Yes (proceed to IVF)	Cystectomy does not improve IVF live birth and may lower yield	Avoid traversing cyst at retrieval; consider prophylaxis
Painful endometrioma, conception not urgent	Possibly	Preservation before surgery	Surgery may relieve pain; protect reserve first	Counsel on AMH fall, especially if bilateral
Suspicious/atypical/rapidly growing cyst	Yes	Preservation if feasible	Exclude malignancy	Do not delay surgery for preservation if cancer suspected
Large cyst obstructing follicle access	Possibly	Individualise	May impair safe retrieval	Weigh access gain against reserve loss
Bilateral endometriomas	Caution—avoid if possible	Strongly favour preservation/ART	High risk to reserve; POI possible	Preserve before any surgery
Recurrent endometrioma after prior surgery	Avoid repeat surgery	Favour ART ± preservation	Repeat surgery compounds reserve loss	Manage symptoms medically where possible
Low ovarian reserve/age ≥38	No	Expedite ART; preserve if delaying	Time and reserve are critical	Refer urgently; do not operate to 'tidy up'
Small, stable, asymptomatic, good reserve, conceiving naturally	No	Expectant ± surveillance	Surgery not indicated	Re-image periodically; reassess if change

**Figure 2:**
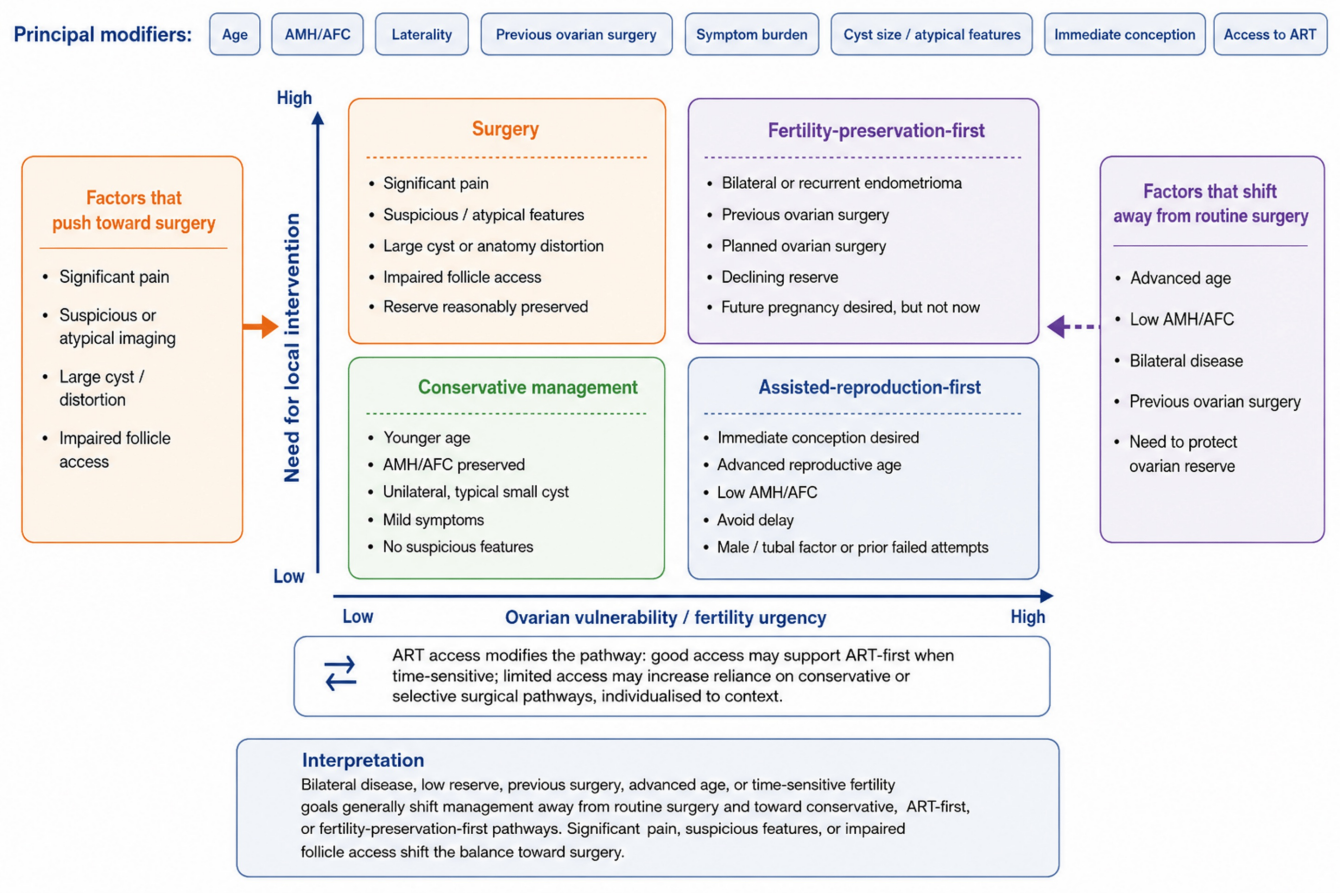
Endometrioma fertility-risk decision matrix The matrix is intended as a counselling aid, not a rigid algorithm. Image Credit: Authors; created using Adobe Illustrator (Adobe Inc., San Jose, California, United States)

Figure 2 translates the above sequence into a practical endometrioma decision matrix. The vertical axis represents the need for local intervention, while the horizontal axis represents ovarian vulnerability and fertility urgency. Clinicians should first assess age, AMH/AFC, laterality, previous ovarian surgery, symptom burden, cyst features, immediate fertility goals, and access to assisted reproductive technology (ART). These modifiers then guide the final pathway: conservative management, surgery, ART first, or fertility preservation first. The matrix should be used as a counselling aid rather than a rigid algorithm.

Because endometrioma surgery is the principal modifiable threat to ovarian reserve in this field, a fertility preservation discussion should precede it whenever feasible, and especially before bilateral or repeat ovarian surgery. The opportunity to bank oocytes or embryos before the reserve is reduced is easily lost if surgery proceeds without it [33,34].

Assisted reproduction

Assisted reproduction is central to managing endometriosis-associated infertility. In selected women with minimal-to-mild disease, patent tubes, adequate reserve, and no significant male factor, intrauterine insemination with ovarian stimulation is a reasonable first-line treatment that is less invasive and less costly than IVF [3,12]. For moderate-to-severe disease, tubal compromise, prolonged infertility, advancing age, diminished ovarian reserve, a significant male factor, or after failed surgery or expectant management, IVF (with ICSI where indicated by sperm parameters) is the more effective route.

An important and reassuring message is that women with endometriosis, including those with endometrioma, generally achieve IVF/ICSI outcomes broadly comparable to those of women without the disease. A large meta-analysis found similar live-birth and clinical-pregnancy rates in women with endometrioma compared with controls, albeit with a modestly lower number of oocytes retrieved and a higher cycle-cancellation rate [9]. Endometriosis may be associated with reduced oocyte yield and, in some studies, lower implantation, but fertilisation rates and per-embryo outcomes are largely preserved, and the disease should not be regarded as a barrier to successful IVF.

Surgical removal of an endometrioma before IVF does not, on current evidence, improve clinical pregnancy or live-birth rates compared with proceeding without surgery and carries the risk of reducing reserve and response [9,10]. Routine pre-IVF cystectomy is therefore not recommended; surgery should be reserved for the specific indications noted above. Standard ovarian stimulation protocols are appropriate; there is no single protocol of proven superiority specific to endometriosis, and choice should be guided by ovarian reserve and prior response. The decision between fresh and frozen embryo transfer should follow general IVF principles rather than the presence of endometriosis per se, though a freeze-all strategy may be considered where coexisting adenomyosis or other factors favour it.

During oocyte retrieval in the presence of an endometrioma, there is a small risk of inadvertent puncture, with theoretical risks of cyst content contamination, abscess formation, and pelvic infection. Many units take care to avoid traversing the endometrioma and consider antibiotic prophylaxis, although the evidence base for routine prophylaxis is limited and practice varies. After surgery, assisted reproduction should not be unduly delayed, particularly in women aged over 35 or with diminished reserve, for whom the cost of waiting is measured in declining oocyte quality. Aligning the timing of any surgery, recovery, and assisted reproduction to avoid wasted reproductive time is one of the most valuable contributions the clinician can make.

Fertility preservation

Fertility preservation has moved from the margins to the centre of endometriosis care, and is a key reason why counselling must begin early. Mature oocyte cryopreservation (vitrification) and embryo cryopreservation are the established techniques, with survival and live-birth outcomes that have improved markedly in the vitrification era [34]. Ovarian tissue cryopreservation, in which cortical tissue is removed, frozen and later re-implanted, has produced live births and could in principle be combined with endometrioma surgery, but in this context it remains largely experimental, is offered in selected centres, and carries the theoretical concern of re-implanting tissue from a diseased ovary [16].

Counselling about fertility preservation should be offered to women at elevated risk of losing ovarian reserve: those with bilateral endometriomas, recurrent endometriomas, previous ovarian surgery, or planned ovarian surgery; those with diminished ovarian reserve; young women with severe disease; and women deferring pregnancy for personal, professional, medical, or social reasons [33,34]. The strongest practical case arises before ovarian surgery, above all before bilateral or repeat surgery, because reserve is most readily protected by banking gametes or embryos while it remains adequate, rather than attempting to recover the position after a fall in AMH. Where a woman is proceeding to IVF for treatment anyway, banking surplus embryos or oocytes from those cycles can serve a dual purpose.

The limitations must be communicated honestly and quantitatively. Outcomes are strongly age-dependent; the chance of a live birth per cryopreserved oocyte falls markedly with age. In studies of elective oocyte vitrification, roughly 8-10 mature oocytes were generally needed to give a reasonable chance of a live birth, and more oocytes were required as age advances [34,35], so women should be encouraged to consider preservation earlier rather than later. The number of oocytes available matters greatly, and women with endometrioma, particularly those who have already had a cystectomy, tend to yield fewer oocytes per cycle, so more than one stimulation cycle (‘oocyte accumulation’) is often required to bank an adequate number, and even then a live birth is not guaranteed [33]. One propensity-matched study found significantly fewer oocytes retrieved in women with endometrioma undergoing preservation than in women without the disease, and reported that young women who had already undergone cystectomy fared worse than non-operated women, an argument for offering preservation before, not after, surgery [33,34]. Cost and access aremajor constraints, and in many health systems and countries, these techniques are simply unavailable or unaffordable, raising real equity concerns. Egg freezing should be presented as a means of improving future options, not as a guarantee of a child.

Recurrence prevention and fertility maintenance

Endometriosis and endometriomas recur, and each recurrence raises the prospect of further surgery that may erode reserve. Maintaining fertility over time, therefore, depends as much on avoiding unnecessary repeat surgery as on any single intervention. Where a woman is not seeking to conceive immediately after surgery, postoperative hormonal suppression, continuous combined or progestin-only therapy, or the LNG-IUS reduces the risk of recurrence and can preserve the interval before she starts trying [24,25]. The aim is to avoid a cycle of recurrence and re-operation that progressively depletes the ovary.

Long-term care includes appropriate surveillance, clear triggers for re-referral (new or worsening symptoms, growth or atypical change in a cyst, a new fertility problem, or a fall in reserve before any planned intervention), and pain management that does not compromise fertility goals. Patients should be educated about when to seek fertility assessment, particularly as they approach their mid-30s or if conception does not occur within the expected interval, so that the decision to act is not left too late. Table 5 provides patient-centred counselling points in clinician-friendly language to support these conversations. The overarching principle is conservatism towards the ovary: protect reserve, intervene only when clearly indicated, and keep assisted reproduction and preservation options in view.

**Table 5:** Counselling points for patients. Statements are intended as adaptable prompts for individualised counselling, not as verbatim scripts. Compiled by the authors from references [1,3,4].

Theme	What to convey to the patient
Age and time	Your age is the single biggest factor in your chance of pregnancy. If you are 35 or older, or if your reserve is reduced, we should not wait too long before seeking specialist help.
The endometrioma itself	An endometrioma can lower your egg reserve even before any operation, and surgery to remove it can lower it further. We will only operate if there is a clear reason to.
Surgery before IVF	Removing an endometrioma before IVF does not usually improve your chance of a baby and may reduce the number of eggs we can collect, so we often proceed to IVF without operating.
Protecting future eggs	If you may need ovarian surgery—especially on both ovaries or a repeat operation—we can discuss freezing eggs or embryos beforehand, while your reserve is better.
Hormonal treatment	Hormone treatments that help your pain work by stopping ovulation, so they are not a fertility treatment while you are trying to conceive. They can be useful after surgery if you are not yet ready to try.
Egg freezing expectations	Freezing eggs improves your future options but is not a guarantee of a baby; success depends mainly on your age and the number of eggs stored, and more than one collection may be needed.
Repeat surgery	We try to avoid repeated operations on the ovaries, because each one can reduce your egg reserve. Where possible we will control symptoms in other ways.
Assisted reproduction	IVF works well for many women with endometriosis, with success rates close to those of women without the condition. We may suggest it sooner if natural conception is unlikely.
Pregnancy	Endometriosis slightly increases some pregnancy risks, so we may watch your pregnancy a little more closely; this is a reason for care, not a reason to avoid pregnancy.
Shared decisions	There is rarely one 'right' answer. We will make these decisions together, taking account of your priorities, and revisit them as your situation changes.

Adolescents and young adults

Endometriosis frequently begins in adolescence, yet recognition is often delayed by years, with severe dysmenorrhoea and absenteeism dismissed or normalised. Earlier recognition matters not only for pain but for the long reproductive horizon ahead. In this group, conservative management is generally preferred: symptom control with hormonal therapy and analgesia, with surgery reserved for diagnostic uncertainty, failure of medical management, or large or complex endometriomas, and always performed with maximal preservation of ovarian tissue [1].

Fertility counselling in adolescents and young adults should be developmentally appropriate, honest about uncertainty, and mindful of the long interval before many will seek pregnancy. Fertility preservation may be considered in severe disease, particularly before extensive or bilateral ovarian surgery, but the evidence in this age group is limited, and the ethical considerations, including consent, the speculative nature of future benefit, and the burden of intervention in a young person, are substantial. Decisions should be individualised, made with the young person and (as appropriate) their family, and ideally involve clinicians experienced in adolescent endometriosis [1].

Deep endometriosis and adenomyosis

Deep (infiltrating) endometriosis, involving structures such as the uterosacral ligaments, rectovaginal septum, bowel, bladder, or ureter, presents particularly difficult fertility decisions. The two broad strategies, primary surgery versus an assisted-reproduction-first approach, each have advocates, and the evidence does not clearly establish the superiority of either for live birth. ESHRE guidelines [1] do not support routine surgery for deep endometriosis solely to improve assisted-reproduction outcomes, while several observational series report favourable postoperative pregnancy rates, including a high proportion of natural conceptions after complete excision of severe rectal disease in appropriately selected younger women [19,20]. These series are encouraging but are subject to selection bias and do not establish that surgery improves live birth across the board.

Two practical points command broad agreement. First, surgery for deep endometriosis affecting bowel, bladder or ureter is complex, carries appreciable morbidity, and should be undertaken in specialist multidisciplinary centres. Second, the decision should be individualised according to symptoms, anatomy, age, ovarian reserve, access to assisted reproduction, and the woman's own priorities, rather than driven by a presumption that all deep disease must be excised before conception is attempted [19,20]. Claims that surgery improves live birth in all cases of deep endometriosis are not supported by the current evidence and should be avoided.

Adenomyosis frequently coexists with endometriosis and is an independent adverse factor for assisted reproduction. Meta-analyses indicate that adenomyosis is associated with a reduced likelihood of clinical pregnancy and implantation and an increased risk of miscarriage after IVF/ICSI [17,18]. Its presence should be sought, particularly in women with otherwise unexplained implantation failure or recurrent loss, and may influence management, for example, consideration of prolonged GnRH-agonist down-regulation or a freeze-all approach; however, the evidence for these strategies remains limited, and they should be applied judiciously rather than routinely [18].

Emerging and investigational management options

Several newer approaches are changing, or may change, the management landscape; clinicians should understand their evidence base and limitations before adopting them, and should distinguish clearly between proven and investigational uses.

Oral GnRH Antagonists and Refined Medical Therapy

Oral, non-peptide GnRH antagonists, elagolix and relugolix, given as a combination tablet, with low-dose oestradiol and norethisterone acetate, have been shown in large phase 3 randomised trials to reduce dysmenorrhoea and non-menstrual pelvic pain compared with placebo, with the add-back combination mitigating hypo-oestrogenic bone loss and allowing longer-term use [22,23]. Their established role is in pain control and in reducing the need for repeated surgery; they are not fertility treatments and, like all ovulation-suppressing therapy, must be stopped before attempting conception. Their relevance to fertility is therefore indirect; better long-term symptom control may reduce the pressure for reserve-damaging surgery in women who wish to preserve future fertility. Continuous dienogest similarly offers effective recurrence prevention, with bone density the main long-term consideration [24].

Reserve-Sparing Endometrioma Treatments

Transvaginal aspiration with ethanol sclerotherapy of endometriomas has attracted interest as a minimally invasive, reserve-sparing alternative to cystectomy in women awaiting IVF. Comparative and observational data suggest that, relative to laparoscopic stripping, sclerotherapy better preserves AMH and may yield more oocytes, with pregnancy rates at least comparable, while older series found that neither resection nor sclerotherapy improved IVF outcomes over no intervention [29,30]. The technique is not standardised, recurrence and ethanol-exposure complications remain concerns, and larger prospective trials are needed before it can be recommended as routine; it is best regarded as a promising option within specialist or trial settings.

Regenerative and Adjunctive Approaches

Intraovarian injection of autologous platelet-rich plasma has been explored as a means of improving ovarian response in women with diminished reserve, including some with prior endometrioma surgery, with small studies reporting modest changes in reserve markers and occasional pregnancies [31]. The evidence is preliminary, uncontrolled in most reports, and insufficient to support routine use; it should be offered only within research protocols. Likewise, in vitro maturation of immature oocytes and other laboratory innovations may eventually expand options for women with compromised ovaries, but remain investigational in this population.

Prediction Tools and Non-invasive Diagnosis

Beyond new treatments, better decision tools are themselves an advance. The EFI provides individualised, validated prediction of non-IVF pregnancy after surgical staging and can now be estimated preoperatively, supporting shared decisions between expectant management, surgery, and assisted reproduction [13,14]. In parallel, efforts to improve non-invasive diagnosis, such as structured ultrasound, MRI and, increasingly, AI-assisted image analysis, aim to shorten diagnostic delay and refine triage, although automated diagnostic tools remain at an early stage and have not replaced expert assessment [15]. Used wisely, such tools may help direct scarce surgical and assisted-reproduction resources to the women most likely to benefit.

Pregnancy outcomes

Although the focus of this review is the achievement of pregnancy rather than its course, women with endometriosis should be counselled that the condition is associated with a modest increase in certain obstetric risks. A large systematic review and meta-analysis reported higher odds of pre-eclampsia and hypertensive disorders, gestational diabetes, placenta praevia, antepartum haemorrhage, caesarean section, preterm birth, small-for-gestational-age infants and, of particular concern, stillbirth and neonatal death, in pregnancies in women with endometriosis [21]. The absolute increases are generally small, the underlying studies are observational and heterogeneous with potential confounding (including by assisted-reproduction use), and the findings should be communicated proportionately so as to inform rather than alarm. Their main implication is that such pregnancies may warrant a slightly heightened index of obstetric vigilance, not that endometriosis should deter women from conceiving.

Global and health system perspective

For an international readership, fertility care in endometriosis must be understood as much through the lens of access as of biology. Diagnostic delay is a global problem, but its consequences are amplified where specialist imaging, high-quality endometriosis surgery, assisted reproduction, and fertility preservation are scarce or unaffordable. The interventions that best protect fertility, including early counselling, careful reserve assessment, conservative surgical decisions, and timely assisted reproduction, are not equally available everywhere, and recommendations must be tiered accordingly.

Several principles travel well across settings. Early counselling and the avoidance of unnecessary or repeated ovarian surgery cost little and protect reserves everywhere. Reserve assessment with AMH or AFC, where available, helps triage women who should be referred without delay, and simple validated tools such as the EFI can structure counselling even in non-specialist clinics [13,14]. Strong referral networks, linking general gynaecology to specialist endometriosis and reproductive medicine centres, allow scarce expertise to be concentrated where it is most needed. Finally, the marked global inequities in access to assisted reproduction, fertility preservation, and newer medical therapies are an ethical as well as a clinical concern, and equitable provision should be advocated as part of comprehensive endometriosis care [3,4].

## Conclusions

In clinical practice, fertility-preserving management of endometriosis should follow a structured sequence: assess fertility intention and age, assess ovarian reserve, define disease phenotype, confirm whether surgery is truly indicated, and then select the pathway that maximises pregnancy potential while minimising avoidable ovarian damage. In women with suspected or confirmed endometriosis, the interventions most likely to maximise pregnancy while protecting ovarian reserve are not single procedures but a coherent, individualised pathway. Fertility should be addressed at the point of suspicion rather than deferred to established infertility, with decisions anchored in age, ovarian reserve, and the duration of infertility.

Ovarian surgery, particularly bilateral or repeat endometrioma cystectomy, is the principal modifiable threat to ovarian reserve and should be reserved for clear indications, with fertility preservation offered beforehand where feasible. Medical therapy controls pain and recurrence but is not a fertility treatment during attempts to conceive, and routine cystectomy before IVF is not supported. Assisted reproduction should be used early where spontaneous conception is unlikely, and oocyte or embryo cryopreservation should be offered selectively with honest, age-calibrated expectations. Newer options, from oral GnRH antagonists to reserve-sparing endometrioma techniques and prediction tools, are promising but should be applied within the limits of current evidence. Equitable access to timely diagnosis, specialist surgery, assisted reproduction, and fertility preservation remains the central challenge on a global perspective.
